# Case report: Vesiculobullous eruption with an atypical IgA deposition pattern in a patient with multiple myeloma—A case report and literature review

**DOI:** 10.3389/fimmu.2023.1121380

**Published:** 2023-02-10

**Authors:** Tong Li, Hongjie Liu, Wei Li

**Affiliations:** ^1^ Department of Dermatology, Rare Diseases Center, West China Hospital, Sichuan University, Chengdu, Sichuan, China; ^2^ Laboratory of Dermatology, Clinical Institute of Inflammation and Immunology, Frontiers Science Center for Disease-related Molecular Network, West China Hospital, Sichuan University, Chengdu, Sichuan, China

**Keywords:** multiple myeloma, autoimmune bullous disease, IgA autoantibodies, monoclonal gammopathy, direct immunofluorescence

## Abstract

Cutaneous vesiculobullous eruptions associated with multiple myeloma (MM) are rare. Although the development of blisters is mostly driven by amyloid deposits of paraproteins in the skin, autoimmunity may play a role. In this study, we report an unusual case of an MM patient with blisters who presented with both flaccid and tense vesicles and bullae. Direct immunofluorescence revealed IgA autoantibody deposits in the basement membrane zone (BMZ) and intercellular space of the epidermis, which displayed an atypical autoantibody deposition pattern. The patient showed rapid disease progression and died during follow-up. We performed a literature review of autoimmune bullous diseases (AIBDs) associated with MM or its precursors and found 17 previously reported cases. Together with the present case, cutaneous involvement of the skin folds was frequently reported, and mucous membranes were barely affected. IgA pemphigus, with consistent IgA monoclonality, was observed in half of the cases. Five patients displayed atypical autoantibody deposition patterns in the skin; the prognosis of these patients appeared to be poorer than that of other patients. We aim to increase our understanding of AIBDs associated with MM or its precursors.

## Introduction

Multiple myeloma (MM) is a malignant disorder of plasma cells that typically manifests as bone pain, anemia, and renal failure in the presence of paraproteins. Skin symptoms are rarely observed in patients with this disorder. Clinically, skin rashes associated with MM can be classified as specific or non-specific. Although cutaneous plasmacytoma is a specific skin condition associated with MM, cutaneous amyloidosis, Sweet’s syndrome, and pyoderma gangrenosum (PG) are common non-specific skin findings in MM patients (1).

Vesiculobullous eruptions are a group of infrequent non-specific dermatoses associated with MM, and their etiology is generally ascribed to the existence of paraproteins (2). Although most cases of vesiculobullous eruptions are induced by amyloid deposits of paraproteins in the skin (3), autoimmunity reportedly plays a role. Autoantibodies against skin antigens can be observed in some patients with MM, resulting in autoimmune bullous diseases (AIBDs). Based on antigen localization, AIBDs can be divided into pemphigus (antigens found in the intercellular space of the epidermis) and pemphigoid [antigens found in the basement membrane zone (BMZ)] diseases. These two entities are considered completely different from each other owing to their distinct clinical features and autoantibody deposition patterns.

In this case report we describe a unique presentation of MM with multiple skin blisters. The patient presented with acute onset of vesiculobullous eruptions. A skin biopsy revealed subepidermal blisters with distinct leukocytoclastic vasculitis; direct immunofluorescence (DIF) revealed IgA autoantibodies in the epidermis and BMZ. Thus, it could not be categorized as pemphigus or pemphigoid disease. The patient showed rapid disease progression and died soon after the diagnosis. In addition, we performed a review of AIBDs associated with MM, or its precursors, and summarized the clinical features, DIF and hematoxylin–eosin (H&E) staining findings, and prognosis. This report aims to increase our understanding of the AIBDs associated with MM or its precursors, to raise clinicians’ awareness of the development of monoclonal gammopathy in patients with IgA bullous dermatosis, and to reinforce the importance of closer follow-up in patients with atypical autoantibody deposition patterns in the skin.

## Case report

**Figure 1:**
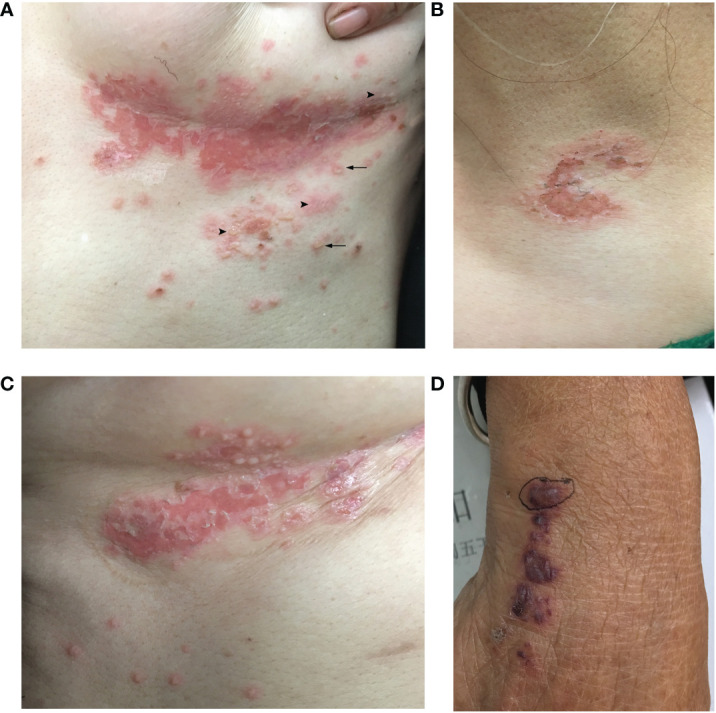
Clinical skin manifestation of the patient. **(A)** Multiple flaccid vesicles, pustules, and erosions on the left inframammary fold. Arrowhead: multiple flaccid vesicles and bullae. Arrow: a few pustules with the appearance of “half–half” blisters. **(B)** Erosion with erythema over the left supraclavicular area. **(C)** Multiple vesicles and erosions on the right inframammary fold. **(D)** Purpura and a hemorrhagic bulla on the right hand.

A 55-year-old Chinese female was referred to the emergency room (ER) at the West China Hospital, Sichuan University. She had complained of an acute onset of pruritic vesiculobullous eruptions on her trunk and extremities 2 weeks before admission, and these eruptions originated primarily from the bilateral inframammary folds. The patient also complained of severe persistent pain in both thighs. Progressive asthenia, fatigue, and weight loss were also observed. She denied having fever, dyspnea, digestive disorders, or any other systemic symptoms. No other significant medical, family, genetic, psychosocial, or past histories were elicited. She had initially been diagnosed with eczema in the local hospital and treated with topical halometasone, but experienced minimal improvement. Physical examination revealed multiple flaccid vesicles, erosions, and scattered pustules on the inframammary folds, the hypochondrium area, and the left supraclavicular area. Some bullae resembled “half–half” blisters (**Figures 1A–C**). The Nikolsky sign for these lesions was positive. Scattered hemorrhagic bullae with negative Nikolsky signs, petechiae, and purpura were observed on her hands and lower legs (**Figure 1D**). No mucous membranes were involved. The muscle strength of both lower limbs was grade 4. No significant muscle tenderness or joint swelling was observed.

**Figure 2:**
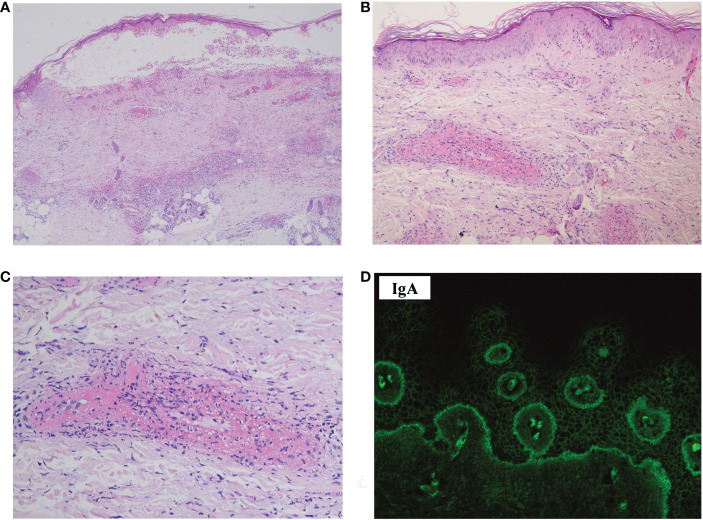
Histopathology, DIF, and immunohistopathology of the skin biopsy sample. **(A)** Hematoxylin–eosin staining showed a subepidermal blister, with perivascular infiltration of inflammatory cells in the dermis (×40). **(B)** Leukocytoclastic vasculitis affecting small-to-medium-sized vessels were observed in the dermis (×100). **(C)** Leukocytoclasis forming nuclear dust and fibrinoid necrosis of the vessels (×200). **(D)** DIF revealed deposits of IgA in the intercellular space of the epidermis and BMZ (×400). DIF, direct immunofluorescence.

The skin biopsy of the hemorrhagic bulla revealed a subepidermal blister and distinct leukocytoclastic vasculitis, primarily affecting small to medium-sized vessels in the dermis (**Figures 2A–C**). Acantholysis was not observed. DIF revealed deposits of IgA autoantibodies in the BMZ and the intercellular space of the epidermis (**Figure 2D**). However, these did not meet the diagnostic criteria for any specific type of IgA-associated bullous disease. IgM and C3 deposits were also detected in the BMZ; IgG deposits were not found (data not shown).

**Figure 3:**
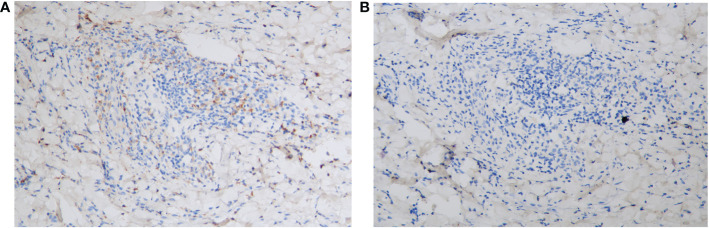
Immunohistopathology of the skin biopsy specimen. **(A)** The monoclonal κ-light chain was positive (×200). **(B)** The monoclonal λ chain was negative (×200).

Laboratory investigations on the day she arrived suggested anemia; her hemoglobin level was 93 g/L, and this steadily decreased to 76 g/L over the next 4 days. Severe impairment of renal function was detected (with a serum creatinine level of 558 μmol/L). Circulating antibodies against either desmoglein (Dsg) 1/3 (Dsg1/Dsg3) or BP180 were not detected by enzyme-linked immunosorbent assay (ELISA) (MBL, Japan). Anti-nuclear antibody (ANA), extractable nuclear antigen (ENA), and anti-neutrophil cytoplasmic antibody (ANCA) panels were negative. Total protein (66.1 g/L; normal: 65.0–85.0 g/L) and globulin (34.1 g/L; normal: 20.0–40.0 g/L) levels were normal. Her IgA level was elevated (3,140.0 mg/L; normal: 836–2900 mg/L), whereas IgG (7.27 g/L; normal: 8–15.5 g/L) and IgM (670.0 mg/L; normal: 700–2,200 mg/L) levels were slightly lower than normal. Monoclonal IgA-κ light chains were detected in the serum by immunofixation electrophoresis. A computed tomography (CT) scan revealed mild pneumonia and multiple nodular lytic lesions in the thorax, axial skeleton, and pelvis. Following bone marrow aspiration and biopsy, the patient was diagnosed with IgA-κ MM. An acute kidney injury associated with MM was also suspected. Further laboratory investigations revealed monoclonal κ-light chains in the dermis, as detected by immunohistopathology (**Figures 3A, B**); amyloids were not detected with Congo red staining (data not shown). During her short stay (i.e., less than 1 week) in the ER, the patient received symptomatic treatments, including vigorous rehydration, electrolyte imbalance correction, and antibiotics (moxifloxacin) for pneumonia. However, after the diagnosis was confirmed, the patient refused a referral to the hematological department and instead asked for a transfer to the local hospital. She died 1 month later.

## Discussion

In this case study we have reported an unusual presentation of vesiculobullous eruptions in a patient with MM. IgA autoantibodies were observed in both the epidermis and the BMZ, which is an atypical deposition pattern. To the best of our knowledge, only one report of MM has previously described a similar deposition pattern in the skin (4), and the IgA autoantibody was seen in the intercellular space of the basal layer, which was not completely the same as in our case.

Varied lesions were observed in this patient. Although the presence of vesicles and erosions suggested a bullous etiology, purpuras over her extremities, indicating an infarctive vascular etiology, were also observed. This may correspond to the histologic findings of distinct leukocytoclastic vasculitis, which have been reported in patients with MM (5). Although a KOH smear test and/or fungal cultures were not taken at the inframammary area, candidiasis and intertrigo were ruled out based on the cutaneous involvement of multiple skin areas and the classic morphology of “half–half” bullae, resembling IgA pemphigus (6). Another differential diagnosis considered was the paraneoplastic pemphigus (PNP) (7). The features of PNP mainly include intractable stomatitis, acantholysis accompanied by interface dermatitis and necrotic keratinocytes, deposition of IgG and C3 in the epidermis and BMZ, and anti-Dsg1/3 and anti-BP180 autoantibodies detected by ELISA (8). Given that our patient did not show the above features of PNP, this diagnosis could be ruled out.

To date, there have been approximately 70 case reports of vesiculobullous lesions associated with MM. The most common dermatosis is bullous amyloidosis, which is caused by cutaneous deposition of light chains originating from neoplastic plasma cells (3). AIBDs associated with MM are rare. We performed a literature review, using PubMed^®^ (National Library of Medicine, Bethesda, MD, USA) and Google Scholar (Google, Mountain View, CA, USA), and found only 17 previously reported cases of AIBDs associated with MM (11/17) or its precursors (6/17), such as monoclonal gammopathy of undetermined significance (MGUS) and smoldering myeloma (9) (**Table 1**). Together with our present case, the median age at which monoclonal gammopathy develops was 60.5 years (range 32–92 years), with approximately equal occurrence in males and females. Among these cases, cutaneous lesions mostly appeared as bullae, vesicles, pustules, and superficial erosions without signs of undermined ulcers. Only one study reported the coexistence of PG (10). In general, lesions can begin several months to years prior to monoclonal gammopathy. Skinfold (i.e., axillae, groin, and inframammary folds) involvement was frequently reported (8/18); mucous membranes were rarely affected (1/18). The IgA type of monoclonal gammopathy, with consistent IgA type of autoantibody deposition in the skin, was observed in 15 out of the 18 cases. Distinct infiltration of neutrophils was found in the skin lesions of all patients with IgA monoclonal gammopathy. Based on the autoantibody deposition pattern, IgA pemphigus was the most frequently reported (i.e., 9/18), followed by pemphigus vulgaris (i.e., 2/18), pemphigus foliaceous (i.e., 2/18), and linear IgA bullous dermatosis (i.e, 1/18). Five cases (4, 10, 16, 20), including ours, demonstrated an atypical IgA deposit pattern in the skin, which did not meet the diagnostic criteria for any specific type of IgA bullous disease. A common feature of these cases was that the patients either responded poorly to treatment (20) or presented with disease progression (4, 16), and all died during follow-up (**Table 1**). Owing to the small sample size, we cannot definitively state that the poor prognosis was related to the unusual autoantibody deposition pattern; further studies are still required.

**Table 1:** Summary of the clinical features of AIBDs associated with MM or its precursors.

Study (first author and year of publication)	Age (years)	Sex	Onset of skin lesions	Distribution of lesions	Mucous membrane involvement	Manifestation of PG	Monoclonal gammopathy subtype	Location of Ig deposits	Dominant inflammatory cells	Diagnosis of lesions	Hematological disorders	Outcome
Wallach, et al., 1982 (10)	92	F	Almost at the same time	Face, limb, trunk, axillary and inguinal folds	No	Yes	IgA-κ	Linear IgA deposition pattern in the epidermis	Neutrophils	SPD	MGUS	Died in hospital
Burrows, et al., 1984 (11)	73	M	2 years prior to MM	Axillae, groin	No	No	IgA-κ	Intercellular IgA in the epidermis	Neutrophils	IgA pemphigus†	MGUS	Controlled
Iwatsuki, et al., 1988 (12)	58	F	8 years prior to MM	Axillae, trunk, and limbs	No	No	IgA	Intercellular IgA in the epidermis	Neutrophils	IgA pemphigus†	MGUS	Controlled
Takata et al., 1994 (13)	59	F	13 years prior to MM	Neck, axillae, groin	No	No	IgA-κ	Intercellular IgA in the epidermis	Neutrophils	IgA pemphigus†	MM	Complete remission
Miyagawa, et al., 1995 (14)	60	F	3 months prior to MGUS	Lateral trunk, dorsal hands, fingers	No	No	IgA-λ	Intercellular IgG, IgA in the epidermis	Neutrophils	Pemphigus foliaceus and IgA pemphigus	MGUS	Controlled
Aste, et al., 2003 (15)	45	M	3 years prior to MGUS	Trunk, extremities, and intertriginous area	No	No	IgA-λ	Intercellular IgA in the epidermis	neutrophils	IgA pemphigus	MGUS	Controlled
Wong et al., 1999 (16)	63	M	5 months prior to MM	Legs, sacrum, buttocks, extensor of the elbow	No	No	IgA-κ	IgA in the dermis	Neutrophils	Undefined	MM	Died 1 year after the first visit
Kurokawa et al., 2005 (17)	62	F	NR	Trunk and lower gingiva	Yes	No	IgG-λ	Intercellular IgG and C3 in the epidermis	Eosinophils	Pemphigus vulgaris	MM	Controlled
Barnadas et al., 2010 (4)	70	M	4 months prior to MM	Thighs and legs	No	No	IgA-λ	Intercellular IgA in the epidermis and BMZ	Neutrophils	Undefined	MM	Died 2 years after the first visit
Szturz et al., 2011 (18)	61	F	6 years prior to MM	Trunk and extremities	No	No	IgA-λ	Intercellular IgA in the epidermis	Neutrophils	IgA pemphigus	MM	Skin lesions improved with hematologic response
Sung et al., 2012 (19)	76	F	27 years prior to MM	Scalp, trunk, and extremities	No	No	IgA-κ	Intercellular IgA in the epidermis	Neutrophils	IgA pemphigus	MM	Controlled
Battistella, et al., 2013 (20)	87	F	Almost at the same time	Legs, trunk, hands, face, scalp	No	No	IgA-λ	IgA in the dermis	Neutrophils	Undefined	MM	Died 3 years after the first visit
Espana et al., 2015 (21)	32	M	2 years prior to MM	Trunk, limbs, and skinfolds	No	No	IgA-κ	Intercellular IgA in the epidermis	Neutrophils	IgA pemphigus	MM	Controlled
Yamaguchi, et al., 2017 (22)	73	M	Almost at the same time	Back, elbow, ankle	No	No	IgA-λ	IgA in the BMZ	Neutrophils	LABD	MGRS	Controlled
Lee et al., 2017 (23)	53	M	2 years prior to MM	Trunk and axilla	No	No	IgA-κ	Intercellular IgG in epidermis	NR	Pemphigus foliaceus-like exanthem	MM	Rapid improvement
Sendrasoa et al., 2018 (24)	55	M	2 months prior to MM	Trunk and scalp	No	No	IgG-λ	Intercellular IgG in the epidermis	Neutrophils and eosinophils	Pemphigus vulgaris	MM	Remission maintained for 12 months
Koga et al., 2022 (25)	42	F	7 years prior to MM	Trunk and lower leg	No	No	IgA-κ	Intercellular IgA in the epidermis	Neutrophils	IgA pemphigus	MM	Controlled
Present case	55	F	Almost at the same time	Trunk, limbs, and inframammary fold	No	No	IgA-κ	Intercellular IgA in the epidermis and BMZ	Neutrophils	Undefined	MM	Died 1 month after the visit

MM, multiple myeloma; BMZ, basement membrane zone; F, female; M, male; NR, not reported; PG, pyoderma gangrenosum; SPD, subcorneal pustular dermatosis; MGUS, monoclonal gammopathy of undetermined significance; MGRS, monoclonal gammopathy of renal significance; LABD, linear IgA bullous dermatosis.

† The cutaneous lesions in these cases were originally described as subcorneal pustular dermatosis (SPD). However, based on the patients’ clinical manifestation and DIF results, the SPD subtype of IgA pemphigus can be considered.

The relationship between autoantibodies against the skin and paraproteins remains unclear. Generally, monoclonal immunoglobulins do not bind to any autologous antigens (18). However, Espana et al. (21) discovered in a patient with IgA pemphigus and IgA-κ MM that the autoantibodies against the skin were monoclonal IgA-κ, suggesting that paraproteins might play a role in the pathogenesis of AIBDs. As our patient presented with similar clinical features, we speculated that the κ-light chains found in the dermis might have the same origin as IgA in the epidermis and BMZ, that is, aberrant plasma cells. The specific antigens in the epidermis or BMZ, against which autoantibodies are formed, remain unclear. In one report, the authors discovered Dsg1/Dsg3 and desmocollin (Dsc) 1–3 autoantibodies for IgA with ELISA-based methods (25). However, the results of ELISAs for anti-Dsg1/Dsg3 and anti-BP180 were negative in our patient. This phenomenon may be explained by the fact that only IgG autoantibodies can be detected using commercial ELISA kits, whereas the main deposits in our patient’s skin were IgA. Nevertheless, in two other studies, despite using multiple laboratory analyses, such as ELISA of Dsg1/Dsg3 and Dsc 1–3 for IgG/IgA and immunoblotting for IgA/IgG, no skin antigens were conclusively identified (13, 21). The major limitation of the present case report is that owing to the rapid progression of the patient’s condition, we were not able to preserve the patient’s serum in time for specialized laboratory tests, such as indirect immunofluorescence, immunoblotting, or immunoprecipitation. Further studies are needed to explore the molecular basis and pathogenesis of AIBDs associated with MM or its precursors.

According to our review, the autoantibodies deposited in the skin, and which display a typical or atypical pattern, are mostly of the IgA type. In addition, concomitant monoclonal gammopathy shows a predilection for the IgA-κ subtype in patients with MM. This implies that IgA-κ monoclonality, rather than other subtypes of monoclonality, may be more closely related to MM-associated IgA dermatosis (25). Histologically, distinct neutrophilic infiltration was observed in all these patients. A recent study found that IgA immune complexes can prime polymorphonuclear neutrophils and lower the threshold for Neutrophil extracellular traps release (NETosis) (26), suggesting that a more active neutrophil chemotactic state may exist in patients with IgA MM.

## Conclusion

The present case and our literature review can increase our understanding of the coexistence of AIBDs and MM or its precursors. Clinicians should be aware of the development of monoclonal gammopathy in patients with IgA bullous diseases. Furthermore, screening for AIBDs in patients with MM and vesiculobullous eruptions is of great importance. Meanwhile, for patients with MM and AIBDs, atypical autoantibody deposition patterns within the skin may require special attention and closer follow-up to evaluate their prognosis.

## Data availability statement

The original contributions presented in the study are included in the article/supplementary material. Further inquiries can be directed to the corresponding author.

## Ethics statement

Ethics review and approval was not required for the study on human participants in accordance with the local legislation and institutional requirements. The patients/participants provided their written informed consent to participate in this study. Written informed consent was obtained from the individuals for the publication of any potentially identifiable images or data included in this article.

## Author contributions

TL was responsible for writing the article. HL was responsible for histopathology support. WL was responsible for article revision. All authors contributed to the article and approved the submitted version.
